# *Zanthoxylum bungeanum* seed oil inhibits tumorigenesis of human melanoma A375 by regulating CDC25A/CyclinB1/CDK1 signaling pathways *in vitro* and *in vivo*

**DOI:** 10.3389/fphar.2023.1165584

**Published:** 2023-04-04

**Authors:** Wanting Wang, Wenwen Pang, Suying Yan, Xiaoli Zheng, Qiurong Han, Yao Yao, Leixin Jin, Chunze Zhang

**Affiliations:** ^1^ Tianjin University of Traditional Chinese Medicine, Tianjin, China; ^2^ Department of Colorectal Surgery, Tianjin Union Medical Center, Tianjin, China; ^3^ Department of Clinical Laboratory, Tianjin Union Medical Center, Tianjin, China; ^4^ The Institute of Translational Medicine, Tianjin Union Medical Center of Nankai University, Tianjin, China; ^5^ Tianjin Institute of Coloproctology, Tianjin, China

**Keywords:** melanoma, *Zanthoxylum bungeanum* seed oil, cell cycle protein, CDC25A/CyclinB1/CDK1 pathway, gut microbes

## Abstract

**Background:** *Zanthoxylum bungeanum* seed oil (ZBSO) is extracted from the seeds of the traditional Chinese medicine *Z. bungeanum* Maxim, which has been shown to have anti-melanoma effects. However, the specific mechanisms are not illustrated adequately.

**Aims:** To further investigate the mechanism by which ZBSO inhibits melanoma and to provide scientific evidence to support ZBSO as a potential melanoma therapeutic candidate.

**Methods:** CCK-8 assays were used to detect the function of ZBSO on A375 cells. Based on transcriptomics analyses, Western blot analysis was applied to determine whether an association existed in ZBSO with the CDC25A/CyclinB1/CDK1 signaling pathway. In addition, RT-qPCR and immunohistochemistry analysis validated that ZBSO has the anti-melanoma effect in a nude mouse xenograft model of human melanoma. Then, 16S rRNA sequencing was used to detect the regulation of gut microbes.

**Results:** Cellular assays revealed that ZBSO could inhibit A375 cell viability by regulating the cell cycle pathway. Further studies presented that ZBSO could constrain CDC25A/CyclinB1/CDK1 signaling pathway *in vitro* and *in vivo* models of melanoma. ZBSO did not produce toxicity in mice, and significantly reduced tumor volume in xenotransplants of A375 cells. Genome analysis indicated that ZBSO successfully altered specific gut microbes.

**Conclusion:** ZBSO inhibited the growth of A375 cells by regulating CDC25A/cyclinB1/CDK1 signaling pathway both *in vitro* and *in vivo*, suggesting that ZBSO may be a novel potential therapeutic agent.

## 1 Introduction

Malignant melanoma, a malignant tumor created by melanocytes, occurs frequently in the skin. The burden of melanoma is estimated to increase to 510,000 new cases and 96,000 deaths by 2040 (Arnold et al., 2022). Although the mortality rate of melanoma has decreased significantly, the prognosis for patients remains gloomy, with a 5-year survival rate of roughly 27% (Siegel et al., 2021). Therefore, it is necessary and urgent to develop novel, potent, and secure anti-melanoma medications.

Chinese herbal medicine has always been an important source of anti-melanoma drug development. Several plant extracts have been shown to have anti-melanoma activity. The ethanolic extract of Huai-Hua-San inhibits STAT3 signaling and demonstrates anti-melanoma properties in cells and mice models. Forsythiae Fructus inhibits B16 melanoma growth *via* MAPK/Nrf2/HO-1-mediated antioxidant and anti-inflammatory activities (Bao et al., 2016; Li et al., 2021). Zanthoxylum bungeanum seed oil (ZBSO) is extracted from the seeds of the traditional Chinese medicine *Zanthoxylum bungeanum* Maxim. ZBSO has a potent anti-inflammatory impact and can accelerate the anti-inflammatory cascade to treat burns in rats, it can also block lung inflammation and effectively treat allergic asthma (Wang et al., 2016; Li et al., 2017). ZBSO was also found to possess antitumor properties. It stimulates autophagy and apoptosis *via* the PI3K/AKT/mTOR signaling pathway in human laryngeal squamous carcinoma cells (Bai et al., 2021). In addition, it inhibits the proliferation of human melanoma cells (A375) (Pang et al., 2019). Its anti-tumor mechanism, however, remains to be elucidated.

Our previous research has reported that ZBSO could significantly inhibit proliferation and promote apoptosis of human melanoma A375 cells *in vitro* by regulating the G1/S cell cycle phase (Pang et al., 2019). According to the previous RNA sequencing data analysis, the cell cycle pathway predominated in the list of ZBSO-regulated pathways, and the key mechanism of the cell cycle pathway might be the transcriptional downregulation of many cell cycle genes, such as cell division cycle 25A (CDC25A) and cyclin-dependent kinases 1 (CDK1) (Pang et al., 2019). However, the ZBSO has had limited efficacy in melanoma, as the molecular mechanism of action remains largely unknown.

Therefore, we continue to study the anti-cancer activities of ZBSO on melanoma cells. By establishing a nude mouse xenograft model of human melanoma, the underlying mechanism of ZBSO was investigated. ZBSO induced similar state changes *in vitro* and demonstrated efficacy *in vivo*, validating the co-culture results. This study provides a mechanistic elucidation of the previously reported cell cycle arrest by ZBSO in melanoma cells, implicating that ZBSO is a novel repurposed drug for cancer therapy.

## 2 Materials and methods

### 2.1 *Zanthoxylum bungeanum* seed oil

A total of 319.05 g *Z. bungeanum* seeds acquired from a traditional Chinese medicine store (Ji’anKang, Chengdu, China, batch number 160801) were pressed to yield 55.0 mL of ZBSO (50.38 g). The obtained ZBSO is decontaminated in a biosafety cabinet by filtration *via* a 0.22 μm membrane, poured into black EP tubes, and kept in a container at −20 °C.

### 2.2 Cell line culture and nude mouse rearing

A375 cells were generously supplied by Yang Jinliang’s team at State Key Laboratory of Biotherapy, Sichuan University, cultured at 37 °C with 5% CO_2_ in Dulbecco’s Modified Eagle’s Medium (DMEM) high sugar medium with 10% (v/v) fetal bovine serum, 100 μg/mL streptomycin, and 100 μg/mL penicillin. A total of 16 male BALB/c nude mice aged 4–6 weeks were purchased from SPF (Beijing) Biotechnology Co. Nude mice were maintained in an SPF animal laboratory at 22°C and 65% humidity, with 12 h of light and 12 h of darkness per day, and all feed, water, and cages were autoclaved before use. Nude mice were adjustably fed for 1 week.

### 2.3 Real-time quantitative reverse transcription PCR (RT-qPCR)

Tumor tissue from nude mice was used for the extraction of RNA. An ultra-micro spectrophotometer, the Nanodrop 2000, was used to quantify the level of RNA in the samples. Reverse transcription was performed using the RevertAid First Strand cDNA Synthesis Kit (Thermo). To inactivate reverse transcriptase, the PCR instrument was kept at 42 °C for 60 min and 70 °C for 5 min. A reaction system was prepared following Supplementary Table S1, and primer designs were listed in Supplementary Table S2. GAPDH was selected as an endogenous control.

### 2.4 Western blot

A375 cells are incubated with 2 mL of 0.45% ZBSO or 0.05% DMSO (Sigma) for 24 h in an incubator. The cells are completely lysed to obtain protein samples for analysis. Proteins were electrophoresed on SDS-PAGE gels (Beyotime Biotechnology) and were transferred to PVDF membranes (Millipore), which were treated with a blocking solution at room temperature for 30 min. PVDF membrane was incubated with the primary antibody (Servicebio) overnight at 4 °C and with the secondary antibodies (Servicebio) for 30 min at room temperature. The results were observed using a gel imaging system (Bio-rad) with β-Actin as an endogenous control.

### 2.5 Construction of a nude mouse xenograft model of human melanoma

Human melanoma A375 cells were digested with 0.25% trypsin (Hyclone, United States). Wash in PBS buffer, centrifuge, and adjust the density of the tumor cell suspension to 1 × 10^8^ cells/mL. Cell suspension (0.1 mL) was injected subcutaneously into the dorsal aspect of the right forelimbs of nude mice sterilized with iodine. The nude mice were maintained until they developed solid tumors. Observe and record daily the survival, body weight, spirit, activity, and growth of tumors in nude mice during a predetermined period. All protocols in this study were approved by the Animal Care and Use Committee of Tianjin Union Medical Center (IRB number: 2023-B01), in compliance with the Guide for the Care and Use of Laboratory Animals published by the US National Institutes of Health (NIH publication no.85-23, revised 1996).

### 2.6 Observation and treatment of xenograft tumor models

When the volume of the tumors in the nude mice reached around 100 mm^3^, the two mice with the largest and two with the smallest tumor volumes were removed. The 12 nude mice were randomly divided into two groups of 6 mice each, and intragastrically administered 3 mL/kg of cold-pressed ZBSO or distilled water daily. Body weights were measured every 2 days and the tumor volume was calculated by using formula V = ab^2^/2 (a is the long diameter, and b is the short diameter). When the xenograft tumor volume of the mice in the control group approached 1,000 mm^3^, the administration was discontinued, blood was drawn from the eyes, and routine blood parameters were analyzed using a veterinary automatic hematology analyzer (Mindray Biomedical Electronics Co., Ltd.). Nude mice were executed using cervical dislocation. The samples were photographed and weighed, and the thymus index, spleen index, and tumor suppression rate were calculated.

$$Thymus\;index=thymus\;weight\;\left(g\right)/mouse\;body\;weight\;\left(g\right)\times 1000$$

$$Spleen\;index=spleen\;weight\;\left(g\right)/mouse\;body\;weight\;\left(g\right)\times 1000$$

$$Tumor\;inhibition\;rate=\left(1-the\;mean\;tumor\;volume\;of\;the\;ZBSO\;group/\;the\;mean\;tumor\;volume\;of\;the\;control\;group\right)\times 100\%$$

### 2.7 HE staining

The paraffin sections were deparaffinized and rehydrated. Stain with hematoxylin for 10 min, differentiate with 1% hydrochloric acid alcohol for 5 s, rinse with tap water, and place the section in warm water at 50°C to blue. After 3 min in 85% ethanol, the sections were stained with eosin for 3 min. Rinse for 5 s with tap water and then dehydrate in gradient ethanol. After sealing the sections with neutral balsam, a digital section scanner is used for image acquisition of the sections. Images are captured at 100× and 400× magnifications to analyze the specific lesion.

### 2.8 TUNEL staining

After deparaffinized and rehydrated, the sections were placed in a moist box. Then 100 μL of Proteinase K working solution (Millipore) was added and the sections were incubated at 37°C for about 25 min before being rinsed in PBS. Add TUNEL reaction mixture (Roche) according to kit instructions. Add 100 μL of DAB (Beijing Zhong Shan-Golden Bridge Biological Technology, Co., Ltd.) and react for about 10 min at room temperature. Stain with hematoxylin for approximately 30 s, then thoroughly rinse with tap water. The sections were taken by a digital section scanner following dehydration in ethanol, transparency in xylene, and sealing with neutral balsam. Three areas were chosen for acquisition at 400×. Calculation of the proportion of apoptotic cells based on Image-Pro Plus 6.0 image analysis system.

### 2.9 Immunohistochemistry

Following deparaffinization and rehydration, the sections were immersed in 3% methanol hydrogen peroxide. The antigenic determinants were repaired by microwave heating in citrate buffer (0.01 mol/L, pH = 6.0). Add goat serum closure solution and react for 20 min at room temperature. Incubate the primary antibody (Beijing Zhong Shan-Golden Bridge Biological Technology, Co., Ltd.) overnight at 4 °C. React for 30 min at room temperature with the secondary antibody. According to the instructions, add horseradish peroxidase, the DAB color development reagent, and hematoxylin. Image acquisition of sections was performed using a digital trinocular camera microscope. Use the Image-Pro Plus 6.0 image analysis system to calculate the average optical density of each image.

### 2.10 Gut microbiological analysis of nude mice

Total DNA was extracted from the collected feces of 12 nude mice. The Illumina MiSeq system was used (Illumina, San Diego, CA, United States) for 16S rRNA sequencing with an optimal sequencing length of 200–450 bp. We utilized the Phylogenetic Investigation of Communities by Reconstruction of Unobserved States (PICRUSt2) software (http://picrust.github.com) to quantify the abundance of secondary functional pathways in the Kyoto Encyclopedia of Genes and Genomes (KEGG) database of diverse microbes and to analyze metabolic pathways by using the MetaCyc database. PICRUSt2 uses one millionth of the sum of the KEGG orthologs (KO) and enzyme commission (EC) abundances for each sample by default. Both the metabolic pathway abundance file and the functional unit abundance file are normalized (Douglas et al., 2020).

### 2.11 Statistical analysis

The experiments were repeated independently at least three times, and the data are expressed as mean ± standard deviation. The data were analyzed using SPSS (version 22.0), and two-by-two comparisons between experimental groups were conducted using the LSD test. Normally distributed data were subjected to the *t*-test and ANOVA to compare differences, with *p <* 0.05 indicating that the differences were significant.

## 3 Results

### 3.1 ZBSO inhibits the expression of apoptosis- and invasion-related proteins

**FIGURE 1:**
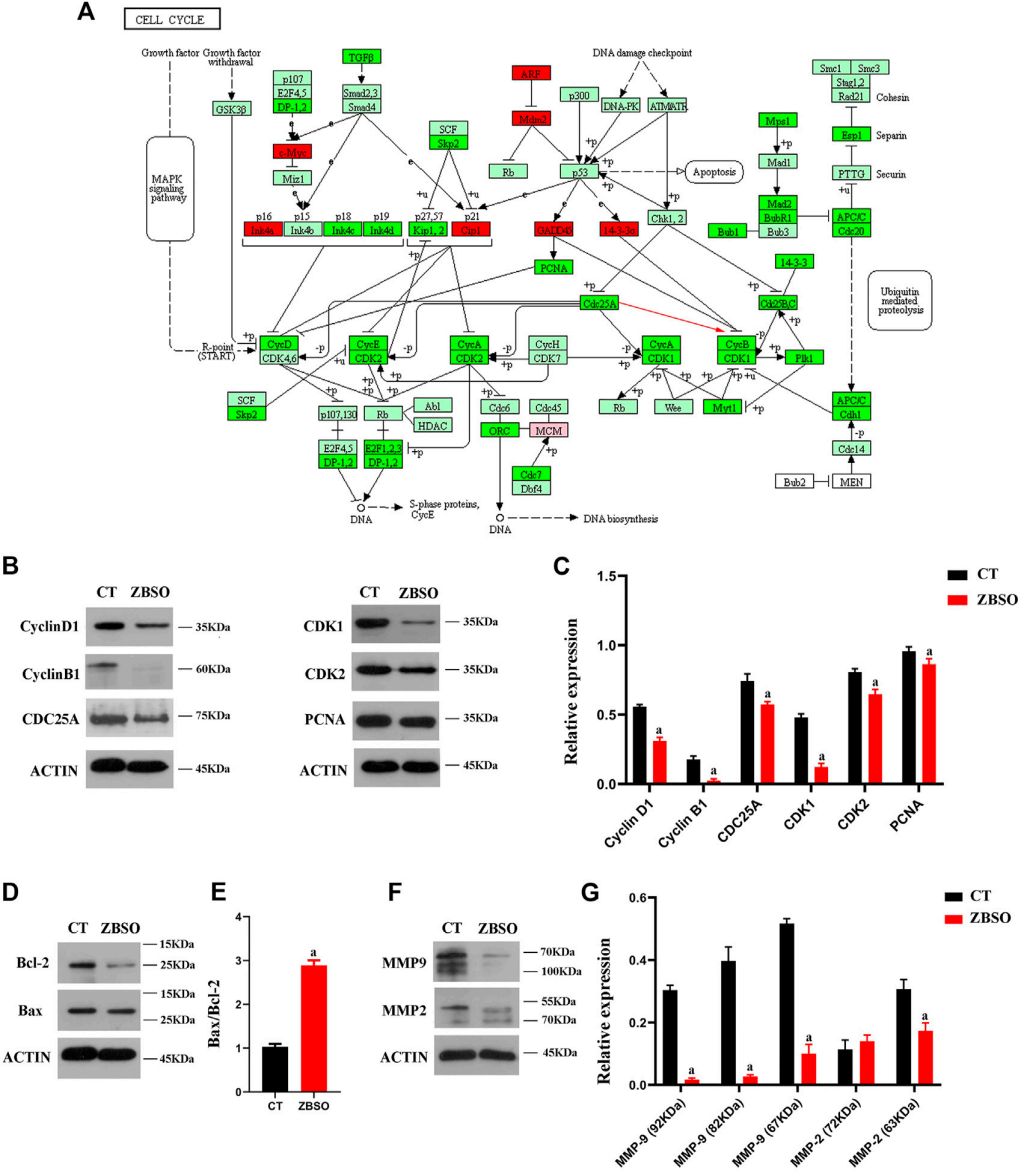
Cell cycle pathways of gene enrichment as well as gene and protein expression in human malignant melanoma A375 cells following treatment with ZBSO. **(A)** Cell cycle pathways of gene enrichment, color represents variations in gene expression, with light green indicating no difference, red indicating upregulation, and bright green indicating downregulation. **(B–G)** Protein expression was measured using Western blot after 24 h of treatment of A375 cells with 0.45% ZBSO. The data in the graph are from the mean ± standard deviation of three independent experiments (^a^ *p* < 0.05 compared with control groups).

The relative expression of Bcl-2 and Bax proteins in human melanoma A375 cells after 24 h of treatment with 0.45% ZBSO is shown in Figures 1D, E. ZBSO treatment resulted in an increase in the Bax/Bcl-2 ratio from 1.03 to 2.89 (*p <* 0.05), which suggests that ZBSO may promote apoptosis in human melanoma A375 cells. A375 cells treated with ZBSO showed a lower relative expression of Matrix Metallopeptidase-9 (MMP-9) zymogen, MMP-9 active enzyme, and Matrix Metallopeptidase-2 (MMP-2) active enzyme (*p <* 0.05) (Figures 1F, G). It appears that ZBSO may inhibit the invasiveness of A375 cells.

### 3.2 ZBSO downregulates the CDC25A/Cyclin B1/CDK1 pathway

To validate the previous RNA sequencing results, we determined the expression levels of relevant proteins in A375 cells after ZBSO treatment. Western blot results confirmed a decrease in Cyclin B1, CDC25A, CDK1, and Proliferating Cell Nuclear Antigen (PCNA) proteins after 24 h of ZBSO treatment (*p* < 0.05) (Figures 1B, C). ZBSO may induce cell cycle arrest in A375 cells by down-regulating PCNA protein expression and the CDC25A/Cyclin B1/CDK1 pathway (Figure 1A).

### 3.3 ZBSO is virtually non-toxic to nude mice

**FIGURE 2:**
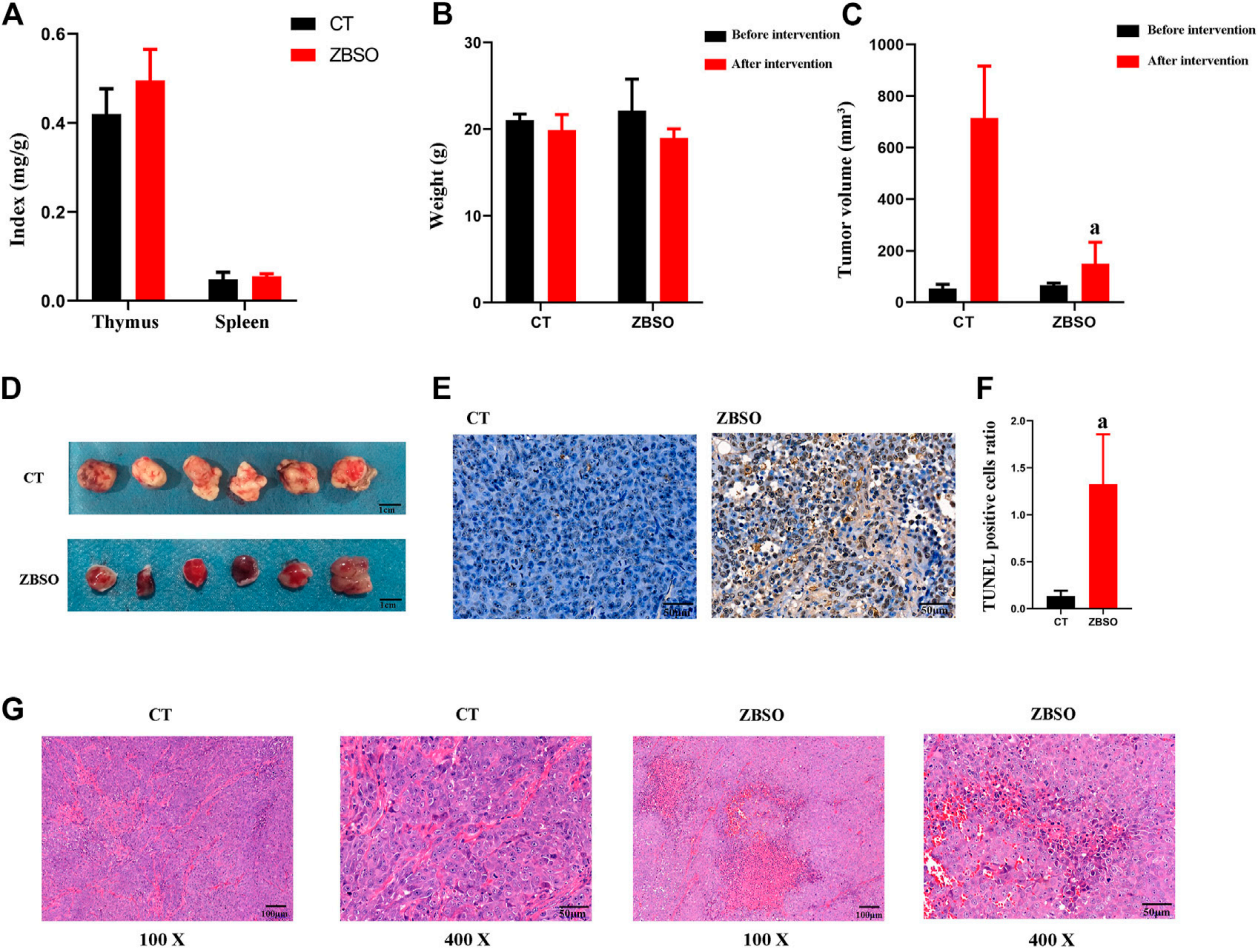
Anti-cancer effects of ZBSO in the nude mouse xenograft model of human melanoma. **(A)** The spleen and thymus index in nude mice. **(B)** The body weight of nude mice before and after the treatment of ZBSO. **(C,D)** The tumor volume of nude mice before and after the treatment with ZBSO. **(E,F)** The TUNEL stained sections of xenograft tumor tissue from control and ZBSO groups of nude mice, at ×400 magnification. Scale bar 50 μm. **(G)** The HE-stained sections of xenograft tumor tissue from control and ZBSO groups of nude mice, at 100 × (Scale bar 100 μm) and 400 × (Scale bar 50 μm) magnification (^a^ *p* < 0.05 compared with control groups).

There was no significant difference between the two groups of nude mice based on routine blood markers (Table 1). Before and after the intervention, the difference in body weight between the two groups of nude mice was not significant (Figure 2B). And the thymus index and spleen index of the two groups of nude mice were not different (Figure 2A).

**TABLE 1:** The effects of ZBSO on blood routine indexes of nude mice.

Blood routine indexes	Control	ZBSO
Red blood cell (10^12^/L)	10.46 ± 0.93	10.54 ± 0.55
Hematocrit (%)	50.92 ± 4.66	49.45 ± 1.80
Mean corpuscular volume (fL)	48.7 ± 1.07	48.22 ± 1.59
Red blood cell volume distribution width (%)	13.1 ± 0.49	12.92 ± 0.27
Hemoglobin (g/L)	169.67 ± 15.84	169.17 ± 10.46
White blood cell (10^9^/L)	1.7 ± 0.49	2.0 ± 0.38
Lymphocyte (10^9^/L)	0.72 ± 0.66	1.05 ± 0.95
Monocytes (10^9^/L)	0.12 ± 0.12	0.15 ± 0.08
Granulocyte (10^9^/L)	0.70 ± 0.76	0.80 ± 0.54
Platelet (10^9^/L)	1,162.83 ± 226.98	1,172.67 ± 273.79
Plateletocrit (%)	0.62 ± 0.06	0.54 ± 0.10
Mean platelet volume (fL)	5.9 ± 0.64	5.5 ± 0.53
Platelet distribution width	16.73 ± 0.44	16.52 ± 0.39

### 3.4 ZBSO inhibits the proliferation of xenograft tumors in nude mice

All nude mice survived and no significant abnormality was observed in the mental, behavioral, or appetite of nude mice in the ZBSO and control groups. It was observed that the xenograft tumor volume of the nude mice in the ZBSO group was significantly smaller than that of the control group (*p <* 0.05) (Figures 2C, D). The tumor inhibition rate of ZBSO was 78.99%, indicating that ZBSO inhibits melanoma *in vivo* significantly.

ZBSO also affected the morphological structure of the xenograft tumor tissue. In HE-stained sections, there was more necrosis, increased eosinophilia in the necrotic areas, the disintegration of tumor cells, blurring of structures, karyorrhexis, karyolysis, increased apoptosis, and a dense arrangement of cells in the tumor growth areas with rounded nuclei and pathological nuclear divisions (Figure 2G). It is clear that ZBSO can induce tumor tissue necrosis in the nude mouse xenograft model of human melanoma.

ZBSO-treated nude mice xenograft tumor tissues exhibited an increase in apoptotic cells when stained with TUNEL (Figures 2E, F). The percentage of apoptotic cells was significantly increased from 0.14 ± 0.06 in the control group to 1.33 ± 0.53 in the ZBSO group (*p <* 0.05), indicating that the ZBSO may promote apoptosis in the nude mouse xenograft model of human melanoma.

**FIGURE 3:**
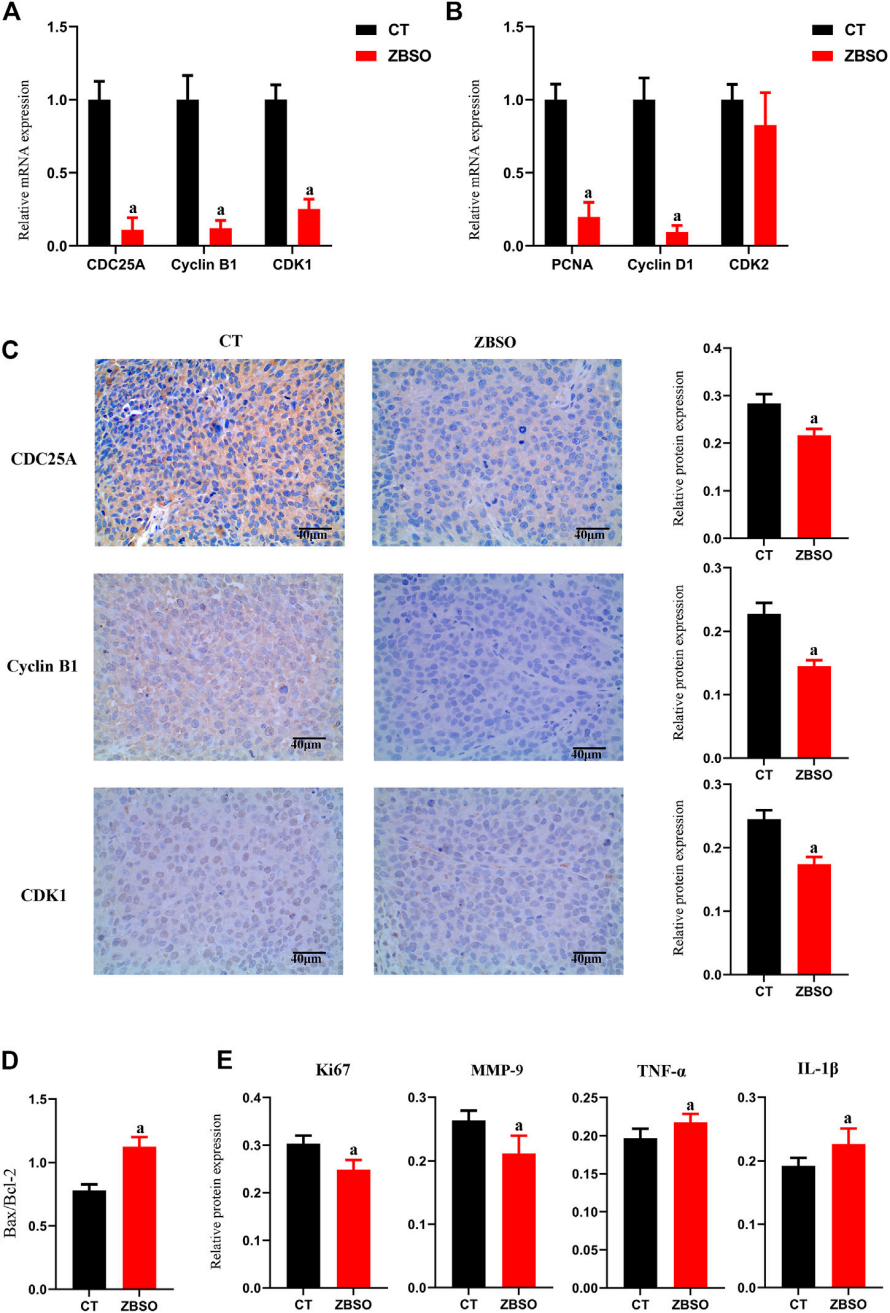
Protein and mRNA expression of xenograft tumor tissue in nude mice. **(A,B)** RT-qPCR analysis of relative mRNA expression levels of cell cycle-related genes in xenograft tumor tissues from nude mice. **(C–E)** Immunohistochemical analysis of target protein expression in xenograft tumor tissues from nude mice. Images of immunohistochemistry were magnified ×400. Scale bar 40 μm (^a^ *p* < 0.05 compared with control groups).

As determined by immunohistochemistry, the ZBSO group had significantly less Ki67 and MMP-9 protein expression than the control group, while the expressions of tumor necrosis factor-α (TNF-α) and Interleukin-1β (IL-1β) were significantly higher (*p <* 0.05) (Figure 3E). The ratio of Bax/Bcl-2 increased significantly from 0.78 to 1.13 in the ZBSO group (*p <* 0.05) (Figure 3D).

### 3.5 ZBSO downregulates CDC25A/CyclinB1/CDK1 pathway to inhibit the growth of transplant tumors in nude mice

ZBSO was shown to cause a significant downregulation of PCNA protein, CDC25A, CyclinB1, and CDK1 *in vitro*. It appears that ZBSO inhibits cancer through the downregulation of PCNA protein and pathways associated with CDC25A/CyclinB1/CDK1. To further prove this hypothesis *in vivo*, we conducted RT-qPCR on xenograft tumor tissue to identify cell cycle-related genes (Figures 3A, B). The relative expression of genes was significantly lower in the ZBSO group, Cyclin B1 was 0.11 ± 0.08, CDC25A was 0.12 ± 0.05, CDK1 was 0.25 ± 0.07, and PCNA was 0.20 ± 0.10 (*p* < 0.05). Furthermore, immunohistochemistry confirmed the expression of these target genes *in vivo*. Consistent with the RT-qPCR results, the outcomes demonstrated that ZBSO was able to inhibit the expression of the aforementioned proteins, with CyclinB1 being from 0.23 ± 0.02 to 0.15 ± 0.003, CDC25A being from 0.28 ± 0.02 to 0.22 ± 0.01, and CDK1 being from 0.24 ± 0.01 to 0.18 ± 0.01 (Figure 3C).

These results provide evidence that ZBSO downregulates PCNA protein expression and the CDC25A/CyclinB1/CDK1 pathway to inhibit the growth of transplant tumors in nude mice.

### 3.6 ZBSO regulates gut microbes in nude mice

**FIGURE 4:**
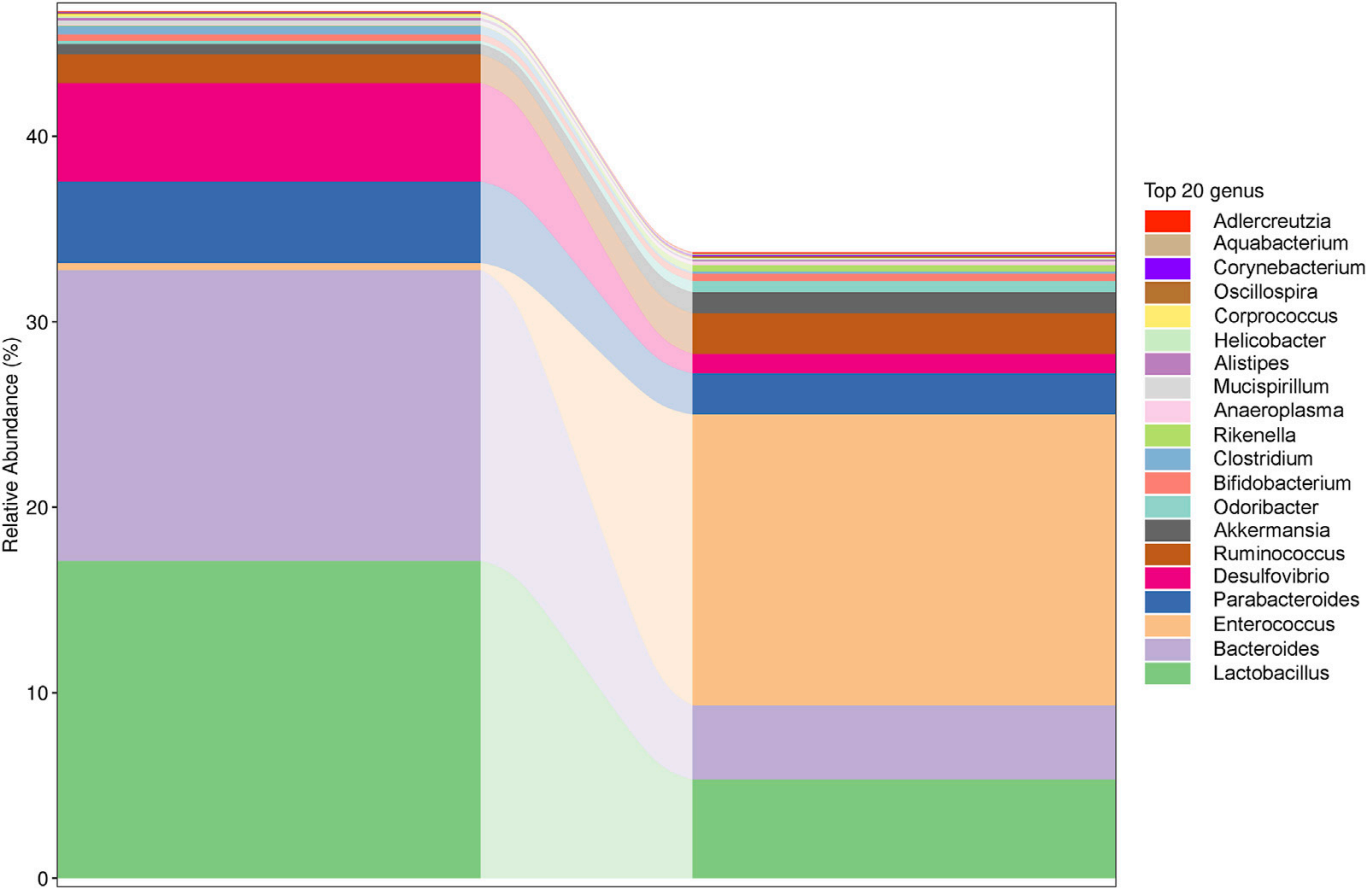
Changes in the relative abundance of the top 20 most significantly different genera.

**FIGURE 5:**
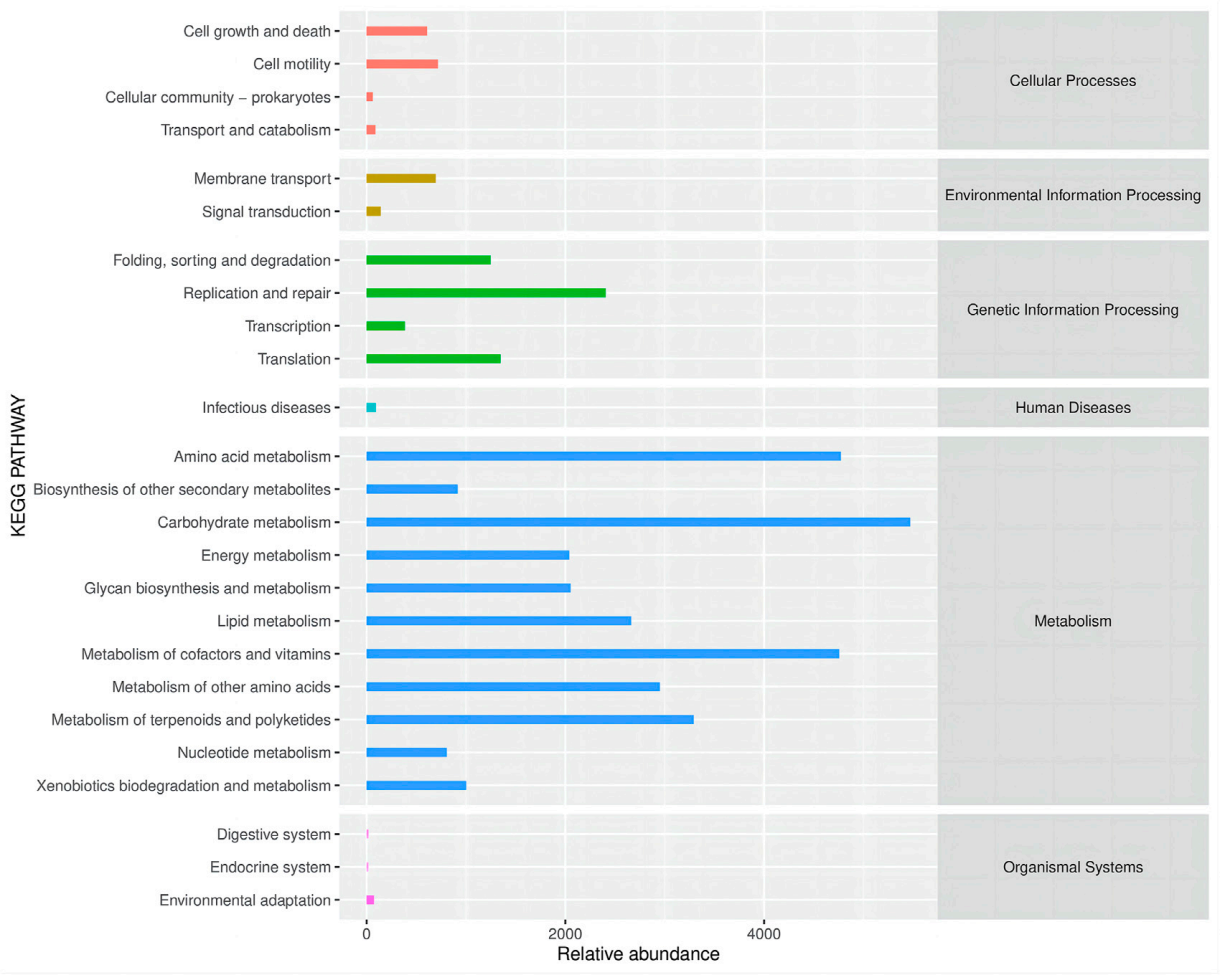
Secondary functional pathways and relative abundance of differential microbial enrichment in the KEGG Biometabolic Pathway Analysis database.

**FIGURE 6:**
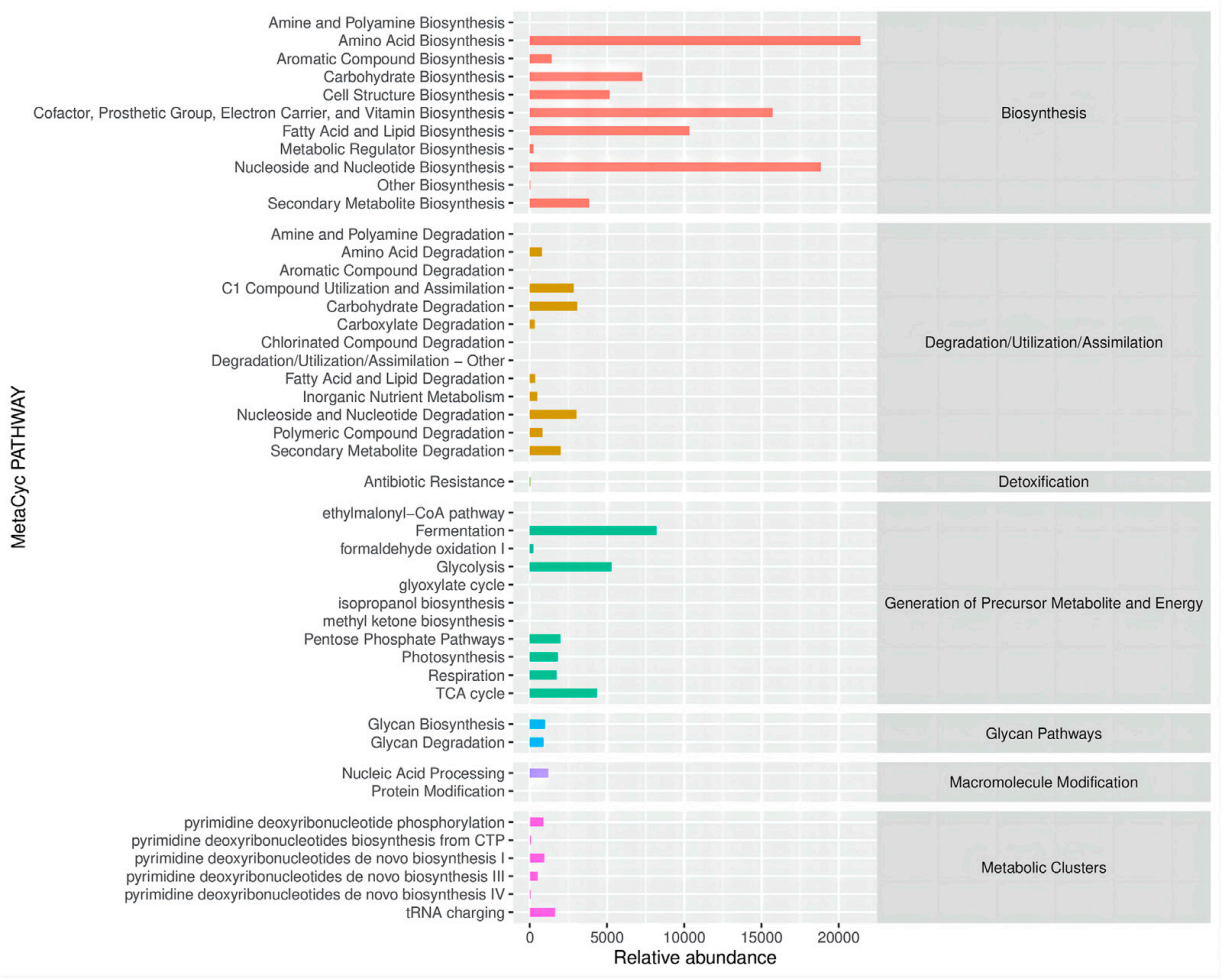
Metabolic pathways and relative abundance of differential microbial enrichment in the MetaCyc database.

Altered gut microbes are critical to tumor development. ZBSO increased the relative abundance of *Enterococcus*, while *Desulfovibrio*, *Parabacteroides*, *Bacteroides*, and *Lactobacillus* decreased (Figure 4). The secondary functional enrichment of KEGG revealed that the functions of the differential gut microbes were focused on cell growth and death in addition to cell motility at the level of cellular processes, primarily carbohydrate metabolism and the metabolism of cofactors and vitamins at the level of metabolism, and primarily replication and repair at the level of gene information processes (Figure 5). Analyses of metabolic pathways revealed that amino acid biosynthetic; cofactor, prosthetic group, electron carrier, and vitamin biosynthetic; nucleoside, and nucleotide biosynthesis were the main biosynthetic pathways of the diverse gut microbes (Figure 6).

## 4 Discussion

As an extract of the natural plant *Zanthoxylum bungeanum* maxim, ZBSO contains numerous bioactive compounds, such as unsaturated fatty acids, phospholipids, and hydroxyandrosterone. ZBSO may serve as a new resource for the exploitation of anti-tumor drugs due to its pharmacological effects such as anti-tumor, anti-inflammatory, immune system regulation, and burns treatment (Beppu et al., 2006; Zhang et al., 2008; Li et al., 2016). Its anti-tumor effect has been reported in many preclinical studies and recently on human laryngeal tumor cells (Bai et al., 2021). The mechanisms, particularly the signaling pathways, remain to be completely elucidated.

In our previous study, ZBSO inhibited the proliferation and invasion and promoted apoptosis of human melanoma A375 cells. In order to further explore the mechanism of ZBSO’s anti-melanoma at a holistic level and to reveal the biological process, high-throughput transcriptome sequencing technology was used to obtain transcript sequence information of A375 cells after ZBSO treatment. Analysis of the data revealed that the cell cycle pathway was the most abundant signaling pathway for differentially expressed genes (Pang et al., 2019). It is suggested that the inhibitory effect of ZBSO on human melanoma A375 cells may be associated with the cell cycle pathway.

Dysregulation of the cell cycle leads to uncontrolled cell proliferation, which plays a key role in the development of malignant melanoma (Lee et al., 2015). Dynamic interactions between cell cycle proteins and their associated cell cycle protein-dependent kinases are responsible for key transitions in the cell cycle (Xu and McArthur, 2016). CDK4, for example, has been well documented that is closely associated with melanoma. In approximately 90% of melanoma cases, the G1-S transition mediated by the CyclinD-CDK4 pathway is dysregulated (Barnaba and LaRocque, 2021). Germline mutations in CDKN2A leading to loss of function and/or CDK4 activating mutations are associated with a 50-fold increased risk of melanoma, according to a study of familial melanoma (Lee et al., 2015). Targeting cell cycle control in advanced melanoma with CDK inhibitors looks to be a promising therapeutic strategy.

As a consequence, it is crucial to determine whether ZBSO inhibits melanoma by regulating cell cycle proteins and the related cell cycle protein-dependent kinases in the cell cycle pathway. Specifically, A375 human melanoma cells were treated with 0.45% ZBSO, and Western blot assays were conducted to determine the levels of several key proteins involved in the cell cycle pathway. A significant decrease in the expression of the proteins CDC25A, CyclinB1, CDK1, and PCNA was observed. PCNA is known as a molecular marker of proliferation because of its role in replication. Three identical molecules of PCNA form a molecular sliding clamp around the DNA double helix (Wang, 2014). It can bind to cyclin-CDK complexes, which may represent an important regulatory mechanism for the recruitment of specific proteins to DNA replication sites (Xiong et al., 1992; Maga and Hubscher, 2003). In the event of PCNA expression being downregulated, it could affect its binding to CyclinB1-CDK1, thereby interfering with DNA replication. CDC25A is a dual specificity protein phosphatase and a positive cell cycle regulator, it activates the cyclin/CDK complexes (Hoffmann et al., 1994). CyclinB1-CDK1 activation is directly affected by the downregulation of CDC25A, resulting in cell cycle arrest. In fact, a variety of cancers have been associated with overexpression of CDC25A, including breast, liver, esophageal, endometrial, and colorectal cancers, as well as non-Hodgkin’s lymphoma (Kristjánsdóttir and Rudolph, 2004; Boutros et al., 2007). Consequently, the downregulation of PCNA protein and the CDC25A/CyclinB1/CDK1 pathway may be the mechanism of ZBSO against melanoma.

*In vivo* test is essential to verify the effectiveness and safety of drugs. A nude mouse xenograft model of human melanoma was constructed to explore the tumor suppression mechanism of ZBSO *in vivo*. By assessing the tumor volume of nude mice, we determined that ZBSO had a tumor suppression rate of 78.99%, with a potent tumor suppressive effect. Immunohistochemical determination of Ki67 and MMP-9 protein expression showed that ZBSO could inhibit tumor proliferation and invasion. The inflammatory cytokines TNF-α and IL-1β are commonly found in the inflammatory environment of various tumors and are often thought to be associated with tumor promotion (Balkwill, 2006; Bent et al., 2018). ZBSO inhibited tumor growth *in vivo* without down-regulating TNF-α or IL-1β. It implies that ZBSO may suppress melanoma independently of TNF-α and IL-1β pathways. The anti-melanoma mechanism of ZBSO was confirmed *in vivo* by RT-qPCR and immunohistochemistry. Consistent with the *in vitro* results, ZBSO downregulated PCNA protein expression and the CDC25A/CyclinB1/CDK1 pathway. In addition, all 12 nude mice survived the experiment and there were no significant differences in body weight, blood index, thymus index, and spleen index between the two groups, which is the first verification of the *in vivo* safety of ZBSO.

Gut microbes are currently garnering a great deal of attention as a significant factor that may impact the development of tumors. Studies have shown that alterations in gut microbes can significantly impact the growth of melanoma *in vitro* and *in vivo* (Li et al., 2019; Hu et al., 2020; Chen et al., 2021). An oral antibiotic depletion of gut microbes significantly reduced the tumor volume in melanoma-bearing mice (Sethi et al., 2018). Additionally, numerous studies have demonstrated that traditional Chinese medicine can modulate intestinal microbes to reduce melanoma growth. *Diosgenin* modulates gut microbes, which enhances the effectiveness of PD-1 antibodies against melanoma (Dong et al., 2018). *Astragalus polysaccharide* inhibits melanoma growth in mice by reshaping the intestinal microenvironment (Ding et al., 2021). It has been suggested that an increased abundance of *Enterococcus* may promote the antitumor activity of checkpoint inhibitor immunotherapy in melanoma patients (Matson et al., 2018; Griffin et al., 2021). *Desulfovibri* enriched in non-responders on anti-programmed cell death protein-1 (anti-PD-1) therapy for melanoma (Pietrzak et al., 2022). An abundance of *Bacteroides* such as *Bacteroides ovatus*, *Bacteroides dorei*, and *Bacteroides massiliensis* is associated with shorter progression-free survival (PFS) in melanoma patients (Brandilyn et al., 2019). The treatment with ZBSO increased the abundance of *Enterococcus* and decreased the abundance of *Desulfovibrio* and *Bacteroides* in the intestines of nude mice. This suggests that changed gut microbes may play a role in the anti-melanoma activity of ZBSO. By inducing cell cycle arrest, *Enterococcus* has been demonstrated to suppress the proliferation of human lymphocytes and gastric cancer MKN74 cells (Lee et al., 2004; Strickertsson et al., 2013). However, it is not clear whether gut microbiome affects melanoma proliferation *via* arresting the cell cycle. It is our intention to further explore the potential mechanisms by which ZBSO affects the gut microbiome to exert an anti-melanoma effect.

Moreover, our work has several limitations, the first one is the insufficient number of nude mouse xenograft models of human melanoma, which will require larger-scale experiments and in-depth research in the future. The second limitation is that we have only examined the anti-melanoma effects of ZBSO *in vivo* using a single dose. In future studies, the multi-dose protocol will be supplemented.

## 5 Conclusion

ZBSO is extracted from the seeds of the traditional Chinese medicine *Z. bungeanum* Maxim. In this study, we confirmed that ZBSO inhibits A375 cell viability by constraining CDC25A/cyclinB1/CDK1 pathway *in vivo* and *in vitro*. Furthermore, ZBSO inhibited tumor growth as well as modulated gut microbes in a nude mouse xenograft model of human melanoma. In conclusion, the data from this study can provide strong evidence that ZBSO could act as a novel potential therapeutic agent for malignant melanoma.

## Data Availability

The datasets presented in this study can be found in online repositories. The names of the repository/repositories and accession numbers can be found in the article/Supplementary Material.
