# Supplementary material for: Zanthoxylum bungeanum seed oil inhibits tumorigenesis of human melanoma A375 by regulating CDC25A/CyclinB1/CDK1 signaling pathways in vitro and in vivo
Source: Front Pharmacol. 2023 Apr 4;14:1165584. doi: 10.3389/fphar.2023.1165584 (PMC10110958; doi:10.3389/fphar.2023.1165584)
Supplement: Supplementary file 1 [file Table1.DOCX]

Supplementary Material

Zanthoxylum bungeanum seed oil Inhibits Tumorigenesis of Human melanoma A375 by regulating CDC25A/CyclinB1/CDK1 Signaling Pathways in vitro and in vivo

Wanting Wang ^†^, Wenwen Pang^†^, Suying Yan, Qiurong Han, Yao Yao, Leixin Jin, Chunze Zhang *

† These authors contributed equally to this work.

* **Correspondence:** Chunze Zhang, [chunze.zhang@nankai.edu.cn](mailto:chunze.zhang@nankai.edu.cn)

# Supplementary Tables

**1.1** Supplementary Table 1 The reaction system of qRT-PCR

| Composition | Volume（μL） |
| --- | --- |
| 2× qPCR Mix | 12.5 |
| 7.5 μM Forward Primer | 1.0 |
| 7.5 μM Reverse Primer | 1.0 |
| Reverse transcription products | 2.5 |
| ddH_2_O | 8.0 |

**1.2** Supplementary Table 2 The sequence of primers of genes

| Gene names | Primer sequences5′-3′) | Length(bp) |
| --- | --- | --- |
| GADPH | F:5′GGAAGCTTGTCATCAATGGAAATC-3′  R:5′-TGATGACCCTTTTGGCTCCC-3′ | 168 |
| Cyclin D1 | F:5′-GCTGGAGCCCGTGAAAAAG-3′  R:5′-ACAGAGGGCAACGAAGGTC-3′ | 298 |
| Cyclin B1 | F:5′-TAAACTTTGGTCTGGGTCGGC-3′  R:5′-CAGGTGCTGCATAACTGGAAGA-3′ | 272 |
| CDC25A | F:5′-TTTGACTCCCCTTCCCTGTGTA-3′  R:5′-GATGTTTCCCAGCAACTGTATGA-3′ | 289 |
| CDK1 | F:5′AAGGGTAGACACAAAACTACAGGTC-3′  R:5′-ATGTACTGACCAGGAGGGATAGA-3′ | 242 |
| CDK2 | F:5′-TGCTCTCACTGGCATTCCTC-3′  R:5′-TGGAGGACCCGATGAGAATG-3′ | 93 |
| PCNA | F:5′-AGCCATATTGGAGATGCTGTTG-3′  R:5′-CTGAGTGTCACCGTTGAAGAGAG-3′ | 230 |
